# Severe acute poisoning among adolescents in Beijing: a 3-year retrospective cohort study at a tertiary referral center (2021–2023)

**DOI:** 10.3389/fpubh.2026.1753377

**Published:** 2026-02-25

**Authors:** Hua Kang, Xiumei He, Lihong Diao, Tiannan Ji, Xiangren Kong, Dong Li, Xiyu He, Biao Xu

**Affiliations:** 1 Emergency Department, The Fifth Medical Center of PLA General Hospital, Beijing, China; 2 Department of Pediatrics, Fifth Medical Center of PLA General Hospital, Beijing, China; 3 State Key Laboratory of Proteomics, Beijing Proteome Research Center, National Center for Protein Sciences (Beijing), Beijing Institute of Lifeomics, Beijing, China

**Keywords:** acute poisoning, adolescents, Beijing, epidemiological survey, pharmaceutical poisoning

## Abstract

**Purpose:**

This study aimed to characterize the epidemiological features, management, and outcomes of acute poisoning among adolescents in Beijing and surrounding areas to guide targeted prevention and clinical intervention.

**Methods:**

A retrospective cohort study was conducted among adolescents (aged 11–18 years) admitted for acute poisoning to the emergency or pediatric department of a tertiary toxicological referral center in Beijing between January 2021 and December 2023. Data on demographics, exposure characteristics, toxic agents, treatments, and clinical outcomes were collected and analyzed.

**Results:**

Among the 915 included cases, there was a significant female predominance (72.5%). Intentional self-harm accounted for the vast majority of incidents (94.5%), with oral ingestion being the primary route (99.5%). A high prevalence of preexisting psychiatric disorders was observed (60.1%). Pharmaceutical poisoning was the most common type (78.1%), followed by pesticides (12.5%). Common interventions included gastrointestinal decontamination (55.7%), extracorporeal elimination (22.3%), and antidote administration (3.1%). While most cases resulted in favorable outcomes (98.7%), adverse outcomes occurred in 1.3% of cases and were primarily associated with herbicide exposure. Logistic regression identified younger age, prehospital interventions, preexisting neuropsychiatric disorders, pesticide exposure, multi-agent mixed exposures, industrial chemical poisoning, and intentional self-harm as significant predictors of hospitalization.

**Conclusion:**

The findings underscore the critical importance of strengthening mental health support for adolescents and implementing stricter controls on the accessibility of psychotropic medications and highly toxic pesticides.

## Introduction

Acute poisoning, including intentional self-harm, remains a leading cause of adolescent mortality and a critical global public health concern (1). According to World Health Organization data, poisoning ranked fifth among unintentional injury-related deaths in children and adolescents aged 0–17 years worldwide in 2008 (2). Beyond fatalities, acute poisoning in this age group frequently necessitates invasive medical interventions, disrupts education, and causes long-term health sequelae, imposing substantial socioeconomic and psychological burdens on families and society (2–4). Comprehensive epidemiological data are urgently needed to guide public health interventions, optimize clinical management, and inform evidence-based prevention strategies.

The epidemiology of adolescent poisoning shows considerable regional variation influenced by socioeconomic, cultural, and policy-related factors (5). However, detailed epidemiological studies on acute adolescent poisoning remain scarce in Beijing, one of the world’s most densely populated metropolises. As a major tertiary referral center for poisoning emergencies in North China, the Fifth Medical Center of Chinese People’s Liberation Army (PLA) General Hospital manages the vast majority of adolescent acute poisoning cases in Beijing, providing representative epidemiological data for this study cohort. We retrospectively analyzed the clinical data of adolescent acute poisoning cases admitted to our center between 2021 and 2023 to characterize the local epidemiological profile. The findings aim to enhance clinicians’ recognition and management of adolescent acute poisoning and to inform evidence-based policies for regional poisoning control systems.

## Materials and methods

### Study design and participants

A retrospective cohort study was conducted utilizing clinical records of adolescents who were admitted for acute poisoning to the Emergency or Pediatric Department of the Fifth Medical Center of the PLA General Hospital from 1 January 2021 to 31 December 2023. Inclusion criteria required patients to be aged 11–18 years with a primary diagnosis of poisoning/drug overdose (ICD-10 codes T36–T65), while exclusion criteria included envenomation (e.g., snakebites), incomplete medical records, or chronic/non-poisoning-related diagnoses.

The protocol was approved by the Medical Ethics Committee (Approval No.: KY-2024-5-72-1), with anonymized data and waived informed consent.

### Data collection and classification

The data extracted from electronic medical records encompassed demographic characteristics (age, sex, residential registration), preexisting comorbidities, exposure details (route, intent, toxic agents, time from exposure to hospital arrival), clinical management (gastrointestinal decontamination, extracorporeal elimination, antidote administration, organ support), treatment setting, emergency department (ED) length of stay (hours), hospitalization duration (days), discharge outcomes, and the number of recurrent poisoning episodes (defined as ≥ 2 distinct acute poisoning events). Regarding exposure intent, cases were defined as intentional self-harm if the emergency record documented a deliberate self-poisoning act; the ‘Abuse’ category encompassed cases of non-therapeutic substance use for psychoactive effects, excluding those classified as intentional self-harm. Toxic agents were categorized as follows: (1) Pharmaceuticals (*Pharmaceutical subclasses were categorized according to their primary therapeutic action and standard pharmacological classification,* including antipsychotics, antidepressants, sedative-hypnotics, neurologic medications, cardiovascular agents, antipyretic-analgesics, other medications, and polypharmacy); (2) Pesticides (herbicides, insecticides, rodenticides, and others); (3) Industrial Chemicals (e.g., methanol, copper sulfate, nitrites); (4) Household Chemicals (e.g., disinfectants, detergents); (5) Ethanol; (6) Other Toxic Agents (e.g., toxic gases, poisonous plants, food-borne toxins); and (7) Multi-category Exposures, referring to co-ingestion of agents from two or more of the above categories (e.g., pharmaceuticals + pesticides + ethanol). Discharge outcomes were stratified as recovered (complete resolution of symptoms), improved (clinically stable for outpatient follow-up), deteriorated (clinical worsening/irreversible complications), death (in-hospital mortality), or other (self-discharge/undocumented transfer), with outcomes dichotomized into favorable (recovered/improved) and unfavorable (deteriorated/death).

### Statistical analysis

Statistical analyses were performed using R 4.3.1. Categorical data are presented as n (%) and compared via χ^2^ or Fisher’s exact tests. Continuous variables are reported as mean ± SD for normal distribution or median (IQR) for non-normal distribution, with group comparisons using *t*-tests/Mann–Whitney *U* (two groups) or ANOVA/Kruskal–Wallis (≥3 groups). Binary logistic regression identified predictors of hospitalization requirement (admission or clinical deterioration/death during ED observation). Pre-specified variables included sex, age, exposure-to-ED time, prehospital interventions, comorbidities, toxic agent category, poisoning intent, gastrointestinal decontamination, and recurrent poisoning. Adjusted odds ratios (aORs) with 95% CIs are reported; *p* < 0.05 (two-tailed) denotes significance.

## Results

### General characteristics

Between January 2021 and December 2023, 971 adolescents with acute poisoning were treated in our emergency or pediatric department. After excluding 56 cases due to incomplete records or non-acute poisoning, 915 cases were included in the analysis (Table 1).

**Table 1:** General characteristics of adolescents with acute poisoning in Beijing and surrounding regions.

Key characteristics	Total *n* (%)	Male *n* (%)	Female *n* (%)	*p*-value
Number of Cases	915 (100.0)	252 (27.5)	663 (72.5)	
Age				<0.001*
11y	12 (1.3)	2 (0.8)	10 (1.5)	
12y	29 (3.2)	4 (1.6)	25 (3.8)	
13y	76 (8.3)	13 (5.2)	63 (9.5)	
14y	156 (17.0)	23 (9.1)	133 (20.1)	
15y	181 (19.8)	65 (25.8)	116 (17.5)	
16y	175 (19.1)	62 (24.6)	113 (17.0)	
17y	145 (15.8)	43 (17.1)	102 (15.4)	
18y	141 (15.4)	40 (15.9)	101 (15.2)	
Time from poisoning to hospital arrival (h)	5.0 (3.0, 9.8)	5.2 (3.0, 9.1)	5.0 (3.0, 10.0)	0.931
Route of exposure				0.001*
Oral	910 (99.5)	247 (98.0)	663 (100.0)	
Inhalation	4 (0.4)	4 (1.6)	0 (0.0)	
Intravenous injection	1 (0.1)	1 (0.4)	0 (0.0)	
Intent of poisoning				<0.001*
Intentional self-harm	865 (94.5)	224 (88.9)	641 (96.7)	
Abuse	34 (3.7)	17 (6.7)	17 (2.6)	
Accidental ingestion	9 (1.0)	6 (2.4)	3 (0.5)	
Environmental	3 (0.3)	3 (1.2)	0 (0.0)	
Unknown	4 (0.4)	2 (0.8)	2 (0.3)	
Preexisting comorbidities				<0.001*
Mental disorders	550 (60.1)	114 (45.2)	436 (65.8)	
Chronic diseases	23 (2.5)	8 (3.2)	15 (2.3)	
None	342 (37.4)	130 (51.6)	212 (32.0)	
Number of recurrent poisoning episodes				0.014*
1 episode	805 (88.0)	233 (92.5)	572 (86.3)	
≥ 2 episodes	110 (12.0)	19 (7.5)	91 (13.7)	
Treatment sitting				0.025*
ED observation	444 (48.5)	118 (46.8)	326 (49.2)	
General ward	377 (41.2)	97 (38.5)	280 (42.2)	
ICU	94 (10.3)	37 (14.7)	57 (8.6)	
Discharge outcome				0.297
Recovered	36 (3.9)	11 (4.4)	25 (3.8)	
Improvement	862 (94.2)	234 (92.9)	628 (94.7)	
Deteriorated	8 (0.9)	2 (0.8)	6 (0.9)	
Death	3 (0.3)	1 (0.4)	2 (0.3)	
Other	6 (0.7)	4 (1.6)	2 (0.3)	

*p < 0.05, ED: Emergency department, ICU: Intensive care unit.

**Figure 1:**
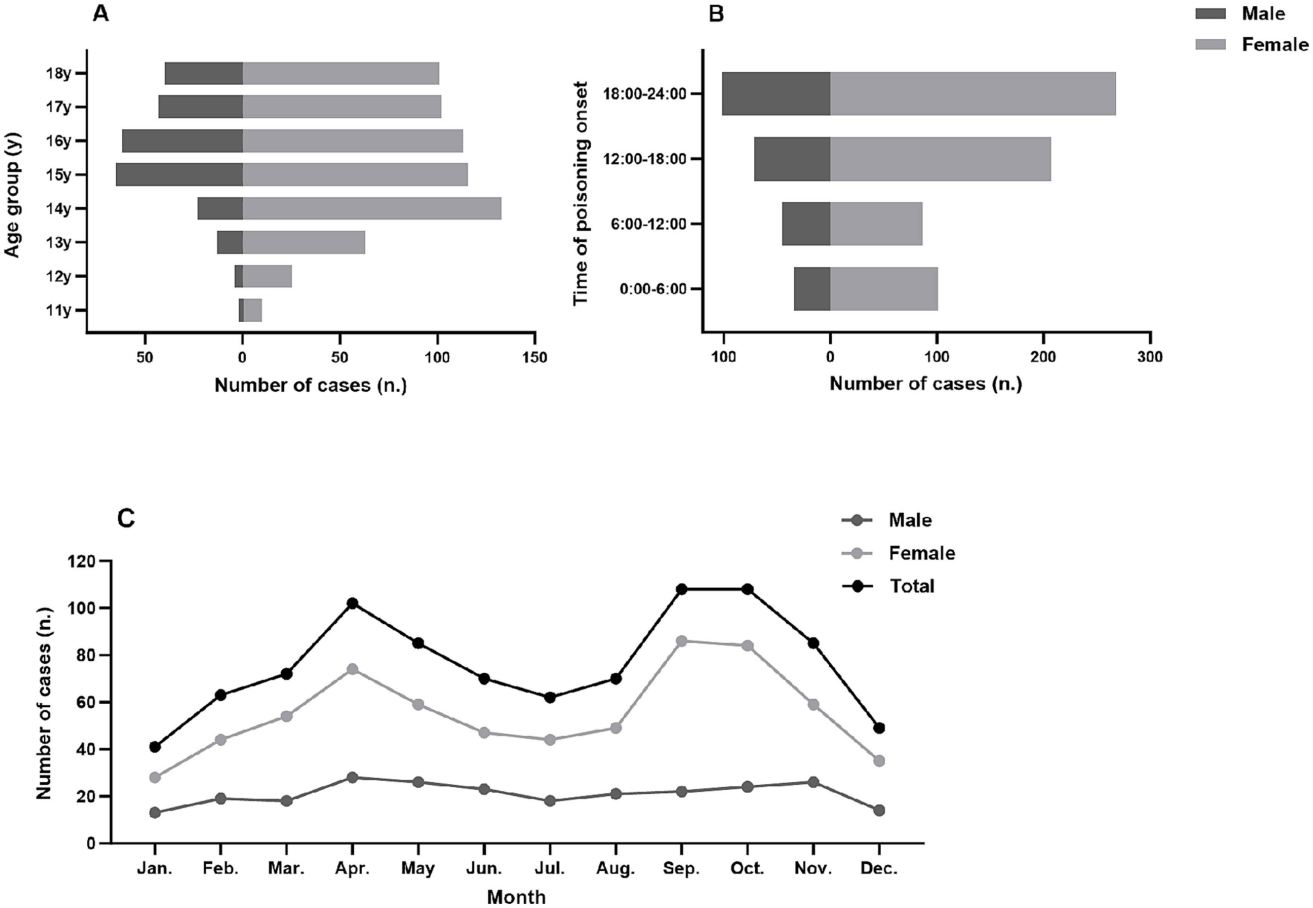
Acute poisoning cases among adolescents in Beijing and surrounding regions, stratified by **(A)** sex, **(B)** time of day, and **(C)** month.

The cohort had a median age of 16 years (IQR: 14–17), peaking at 15 years. Males (*n* = 252) were older than females (*n* = 663) [median 16 years (IQR: 15–17) vs. 15 years (IQR: 14–17); *p* < 0.001]. The highest number of cases occurred among 14-year-old females (20.1%) and 15-year-old males (25.8%) (Figure 1A). Oral ingestion constituted the primary exposure route (99.5%), followed by inhalation (*n* = 4) and intravenous injection (promethazine hydrochloride; *n* = 1). Intentional self-harm accounted for 865 cases (94.5%), while substance abuse represented 34 cases (3.7%). Neuropsychiatric comorbidities were present in 550 patients (60.1%), and 110 patients (12.0%) had recurrent poisoning episodes. Clinic outcomes were as follows: Recovered (*n* = 36, 3.9%), Improved (*n* = 862, 94.2%), Deteriorated (*n* = 8, 0.9%), and Death (*n* = 3, 0.3%).

Sex-based differences were significant in age distribution, exposure route, poisoning intent, preexisting comorbidities, multiple poisoning encounters (all above *p* < 0.001), and treatment setting (*p* = 0.025), but not in time from exposure to hospital presentation (*p* = 0.931) or discharge outcomes (*p* = 0.297).

### Residential registration distribution, temporal patterns, and seasonal variations

Among adolescent poisoning cases, urban residents accounted for 539 cases (58.9%), rural residents for 207 cases (22.6%), and undocumented status for 169 cases (18.5%; Table 2). Urban residents showed higher female poisoning cases (76.8%, 414/539) compared to rural areas (64.3%,133/207; *p* < 0.001).

**Table 2:** Distribution of acute poisoning cases among adolescents, stratified by toxic agent, sex, residence, and season.

Toxic agent category	Sex *n*. (%)		Residential registration *n*. (%)			Season *n*. (%)			
	Male *n* = 252	Female *n* = 663	Urban *n* = 539	Rural *n* = 207	Unknown *n* = 169	Spring *n* = 259	Summer *n* = 202	Autumn *n* = 301	Winter *n* = 153
Pharmaceuticals	156 (21.8)	559 (78.2)	455 (63.6)	123 (17.2)	137 (19.2)	203 (28.4)	164 (22.9)	231 (32.3)	117 (16.4)
Antipsychotics	36 (20.1)	143 (79.9)	117 (65.4)	30 (16.8)	32 (17.9)	51 (28.5)	42 (23.5)	59 (33.0)	27 (15.1)
Antidepressants	30 (19.2)	126 (80.8)	104 (66.7)	24 (15.4)	28 (17.9)	31 (19.9)	37 (23.7)	57 (36.5)	31 (19.9)
Polypharmacy	34 (23.8)	109 (76.2)	90 (62.9)	26 (18.2)	27 (18.9)	42 (29.4)	28 (19.6)	53 (37.1)	20 (14.0)
Sedative-hypnotics	20 (26.7)	55 (73.3)	45 (60.0)	13 (17.3)	17 (22.7)	23 (30.7)	21 (28.0)	21 (28.0)	10 (13.3)
Other medications	13 (22.8)	44 (77.2)	33 (57.9)	12 (21.1)	12 (21.1)	23 (40.4)	10 (17.5)	16 (28.1)	8 (14.0)
Antipyretic analgesics	6 (13.0)	40 (87.0)	29 (63.0)	9 (19.6)	8 (17.4)	16 (34.8)	8 (17.4)	11 (23.9)	11 (23.9)
Neurological agents	8 (26.7)	22 (73.3)	17 (56.7)	7 (23.3)	6 (20.0)	11 (36.7)	10 (33.3)	4 (13.3)	5 (16.7)
Cardiovascular agents	9 (31.0)	20 (69.0)	20 (69.0)	2 (6.9)	7 (24.1)	6 (20.7)	8 (27.6)	10 (34.5)	5 (17.2)
Pesticides	50 (43.9)	64 (56.1)*	34 (29.8)	67 (58.8)*	13 (11.4)	34 (29.8)	23 (20.2)	41 (36.0)	16 (14.0)
Herbicides	31 (40.3)	46 (59.7)	24 (31.2)	49 (63.6)	4 (5.2)	21 (27.3)	13 (16.9)	32 (41.6)	11 (14.3)
Insecticides	12 (52.2)	11 (47.8)	6 (26.1)	11 (47.8)	6 (26.1)	10 (43.5)	7 (30.4)	5 (21.7)	1 (4.3)
Rodenticide	7 (53.8)	6 (46.2)	4 (30.8)	7 (53.8)	2 (15.4)	3 (23.1)	3 (23.1)	3 (23.1)	4 (30.8)
Multi-category mixed	12 (36.4)	21 (63.6)	19 (57.6)	7 (21.2)	7 (21.2)	9 (27.3)	6 (18.2)	10 (30.3)	8 (24.2)
Industrial chemicals	10 (58.8)	7 (41.2)*	10 (58.8)	4 (23.5)	3 (17.6)	5 (29.4)	2 (11.8)	5 (29.4)	5 (29.4)
Household chemicals	5 (50.0)	5 (50.0)*	7 (70.0)	2 (20.0)	1 (10.0)	2 (20.0)	3 (30.0)	3 (30.0)	2 (20.0)
Ethanol	12 (75.0)	4 (25.0)*	6 (37.5)	3 (18.8)	7 (43.8)	2 (12.5)	4 (25.0)	6 (37.5)	4 (25.0)
Other toxic agents	7 (70.0)	3 (30.0)*	8 (80.0)	1 (10.0)	1 (10.0)	4 (40.0)	0 (0.0)	5 (50.0)	1 (10.0)

* *p* < 0.05 for the major category of poisoning compared to pharmaceuticals.

Poisoning incidents peaked in autumn (n = 301, 32.9%) and were lowest in winter (n = 153, 16.7%; Table 2). September and October had the highest monthly cases (108 each, 11.8%), while December had the lowest (n = 49, 5.4%; Figure 1C). Most cases (40.4%) occurred between 18:00–24:00, with fewest from 06:00–12:00 (14.4%; Figure 1B). No sex-based differences existed in seasonal, monthly, or diurnal patterns (all *p* > 0.05).

### Toxic agent distribution

Among 915 adolescent acute poisoning cases, pharmaceuticals were most frequent (*n* = 715, 78.1%), followed by pesticides (*n* = 114, 12.5%) and multi-category exposures (*n* = 33, 3.6%). Industrial chemicals (*n* = 17, 1.9%), ethanol (*n* = 16, 1.7%), household chemicals and other toxic agents (*n* = 10 each, 1.1%) comprised the remainder (Table 2).

For pharmaceutical poisonings, antipsychotics predominated (*n* = 179, 25.0%), followed by antidepressants (*n* = 156, 21.8%) and polypharmacy (*n* = 143, 20.0%). Sedative-hypnotics (*n* = 75, 10.5%), other medications (*n* = 57, 8.0%), antipyretic analgesics (*n* = 46, 6.4%), and neurological (*n* = 30, 4.2%) and cardiovascular agents (*n* = 29, 4.1%) followed. Among pesticides (*n* = 114), herbicides were most common (*n* = 77, 67.5%), followed by insecticides (*n* = 23, 20.2%) and rodenticides (*n* = 13, 11.4%).

The most prevalent agents were sertraline (15.0%), lorazepam (13.2%), quetiapine (11.7%), oxazepam (6.3%), and valproic acid (6.2%). Unfavorable outcomes were linked to diquat (*n* = 5), paraquat (*n* = 3), chlorfenapyr, nitrite, and colchicine (*n* = 1 each).

Compared to pharmaceutical poisonings, significant sex-based differences were observed in cases involving pesticides, industrial chemicals, household chemicals, ethanol (all *p* < 0.001), and other toxicants (*p* = 0.002). Significant urban–rural differences were found for pesticides (*p* < 0.001), while no significant seasonal variations were observed (Table 2).

### Prehospital and in-hospital management

Following exposure, 382 adolescents (41.7%) presented more than 6 h after exposure, while 533 (58.3%) arrived within 6 h; among these, 244 patients (26.7%) presented within 3 h. After initial emergency management, 377 patients (41.2%) were admitted to the general ward, 94 (10.3%) to the ICU, and 444 (48.5%) were placed under ED observation.

Treatment varied significantly across toxicant categories (*p* < 0.001). Pesticide (*p* < 0.001) and industrial chemical poisonings (*p* = 0.029) had higher admission rates than pharmaceutical cases, while multi-category (*p* = 0.036) and ethanol poisonings (*p* = 0.020) showed lower rates. Within pharmaceutical subclasses, Cardiovascular agent exposures had higher observation rates than antipsychotics (*p* = 0.005). Among pesticides, herbicide poisonings showed higher hospitalization rates than insecticide (*p* = 0.018) or rodenticide exposures (*p* < 0.001) (Table 3).

**Table 3:** Clinical management of adolescent acute poisoning cases in Beijing and surrounding regions, by toxic agent category.

Toxic agent category	Treatment site (%)			Treatment measurement				
	EM Room *n* = 444	General Ward *n* = 377	ICU *n* = 94	Gastrointestinal decontamination *n* = 510	Antidote administration *n* = 28	Extracorporeal elimination *n* = 204	Mechanic ventilation *n* = 41	Vasoactive agents *n* = 33
Pharmaceuticals	368 (51.5)	295 (41.3)	52 (7.3)	393 (55.0)	11 (1.5)	133 (18.6)	26 (3.6)	27 (3.8)
Antipsychotics	83 (46.4)	81 (45.3)	15 (8.4)	111 (62.0)	3 (1.7)	37 (20.7)	8 (4.5)	5 (2.8)
Antidepressants	90 (57.7)	57 (36.5)	9 (5.8)	77 (49.4)^**†**^	1 (0.6)	24 (15.4)	5 (3.2)	2 (1.3)
Polypharmacy	67 (46.9)	64 (44.8)	12 (8.4)	85 (59.4)	1 (0.7)	31 (21.7)	7 (4.9)	13 (9.1)^**†**^
Sedative-hypnotics	42 (56.0)	32 (42.7)	1 (1.3)	44 (58.7)	3 (4.0)	5 (6.7)^**†**^	0 (0.0)	0 (0.0)
Other medications	32 (56.1)	20 (35.1)	5 (8.8)	28 (49.1)	0 (0.0)	12 (21.1)	1 (1.8)	1 (1.8)
Antipyretic analgesics	23 (50.0)	23 (50.0)	0 (0.0)	16 (34.8)^**†**^	3 (6.5)	7 (15.2)	0 (0.0)	0 (0.0)
Neurological agents	13 (43.3)	13 (43.3)	4 (13.3)	14 (46.7)	0 (0.0)	12 (40.0)^**†**^	2 (6.7)	0 (0.0)
Cardiovascular agents	18 (62.1)	5 (17.2)	6 (20.7) ^**†**^	18 (62.1)	0 (0.0)	5 (17.2)	3 (10.3)	6 (20.7)^**†**^
Pesticides	22 (19.3)	53 (46.5)	39 (34.2) ^*^	75 (65.8)^*^	12 (10.5)^*^	58 (50.9)^*^	12 (10.5)^*^	4 (3.5)
Herbicides	9 (11.7)	33 (42.9)	35 (45.5)	53 (68.8)	0 (0.0)	54 (70.1)	8 (10.4)	2 (2.6)
Insecticides	7 (30.4)	12 (52.2)	4 (17.4)‡	17 (73.9)	9 (39.1)^**‡**^	3 (13.0)^**‡**^	4 (17.4)	2 (8.7)
Rodenticide	5 (38.5)	8 (61.5)	0 (0.0)^**‡**^	4 (30.8)^**‡**^	3 (23.1)^**‡**^	1 (7.7)^**‡**^	0 (0.0)	0 (0.0)
Multi-category mixed	24 (72.7)	9 (27.3)	0 (0.0) ^*^	19 (57.6)	1 (3.0)	5 (15.2)	1 (3.0)	1 (3.0)
Industrial chemicals	4 (23.5)	10 (58.8)	3 (17.6) ^*^	14 (82.4)^*^	3 (17.6)^*^	8 (47.1)^*^	2 (11.8)	1 (5.9)
Household chemicals	7 (70.0)	3 (30.0)	0 (0.0)	5 (50.0)	0 (0.0)	0 (0.0)	0 (0.0)	0 (0.0)
Ethanol	14 (87.5)	2 (12.5)	0 (0.0) ^*^	2 (12.5)^*^	0 (0.0)	0 (0.0)	0 (0.0)	0 (0.0)
Other toxic agents	5 (50.0)	5 (50.0)	0 (0.0)	2 (20.0)^*^	1 (10.0)	0 (0.0)	0 (0.0)	0 (0.0)

*p < 0.05 for the major category of poisoning compared to pharmaceuticals; †*p* < 0.05 for the pharmaceuticals poisoning subtype compared to Antipsychotics; ‡*p* < 0.05 for the pesticides poisoning subtype compared to Herbicides.

### Therapeutic interventions

Therapeutic interventions primarily included gastrointestinal decontamination (gastric lavage/emesis; 55.7%, *n* = 510), extracorporeal elimination (e.g., hemodialysis/hemoperfusion; 22.3%, *n* = 204) and antidote administration (3.1%, *n* = 28; Table 3). Organ support comprised mechanical ventilation (4.5%, *n* = 41) and vasoactive agents (3.6%, *n* = 33).

Relative to pharmaceutical poisonings, pesticide exposures showed higher rates of gastrointestinal decontamination (*p* = 0.039), antidote administration (*p* < 0.001), extracorporeal elimination (*p* < 0.001), and mechanical ventilation (*p* = 0.002). Industrial chemical poisonings also demonstrated higher rates of these interventions (decontamination: *p* = 0.027; antidote: *p* = 0.003; extracorporeal elimination: *p* < 0.001), whereas ethanol exposures showed lower decontamination rates (*p* = 0.002) as compared with pharmaceuticals.

Among pharmaceuticals, compared to antipsychotics, sedative-hypnotics required less extracorporeal elimination (*p* = 0.011), whereas neurologic agents required more (*p* = 0.038). Similarly, intentional polypharmacy (*p* = 0.028) and cardiovascular agents (*p* = 0.001) were associated with increased use of vasoactive agents.

Within pesticide subcategories compared with herbicides, insecticide and rodenticide poisonings showed increased antidote administration (*p* < 0.001 and *p* = 0.002, respectively) but decreased extracorporeal elimination (both *p* < 0.001). Rodenticides also reduced gastrointestinal decontamination (*p* = 0.013).

### Prognostic indicators

Treatment duration differed significantly across toxicant categories (*p* < 0.001). Pesticide poisonings required the longest treatment (median 7.7 days; IQR 3.1–12.4), followed by industrial chemicals (median 4.5 days; IQR 1.5–7.6) and pharmaceuticals (median 2.0 days; IQR 0.5–5.1). Ethanol intoxication had the shortest duration (median 0.2 days; IQR 0.2–0.3; Table 4). Compared with pharmaceuticals, pesticide poisonings showed prolonged durations (*p* < 0.001), whereas ethanol intoxication was shorter (*p* < 0.001) (Table 4).

**Table 4:** Prognosis of acute poisoning in adolescents from Beijing and surrounding regions, by toxic agent category.

Toxic agent category	Treatment duration			Discharge outcome					Recurrent Poisoning Episodes *n*. (%), *n* = 110
	Emergency observation (h), *n* = 444	Hospitalization Length (d), *n* = 377	Total (d), *N* = 94	Covered *n*. (%) *n* = 36	Improved *n*. (%) *n* = 862	Deteriorated *n*. (%) *n* = 8	Death *n*. (%) *n* = 3	Others n. (%) *n* = 6	
Pharmaceuticals	10.7 (6.3,15.6)	5.0 (4.0, 6.0)	2.0 (0.5, 5.1)	30 (4.2)	679 (95.0)	1 (0.1)	0 (0.0)	5 (0.7)	100 (14.0)
Antipsychotics	10.6 (6.0, 15.1)	5.0 (4.0, 6.0)	3.0 (0.6, 5.2)	5 (2.8)	173 (96.6)	0 (0.0)	0 (0.0)	1 (0.6)	26 (14.5)
Antidepressants	11.1 (7.2, 16.0)	5.0 (3.0, 6.0)	1.1 (0.5, 4.9)	8 (5.1)	147 (94.2)	0 (0.0)	0 (0.0)	1 (0.6)	21 (13.5)
Polypharmacy	11.9 (7.4, 16.4)	5.0 (3.8, 7.0)	2.8 (0.6, 5.5)	9 (6.3)	132 (92.3)	0 (0.0)	0 (0.0)	2 (1.4)	25 (17.5)
Sedative-hypnotics	9.3 (5.4, 13.1)	4.0 (3.0, 5.0)	0.8 (0.4, 4.0)	2 (2.7)	72 (96.0)	0 (0.0)	0 (0.0)	1 (1.3)	7 (9.3)
Other medications	10.5 (6.7, 13.5)	5.0 (3.0, 5.0)	1.0 (0.4, 4.4)	1 (1.8)	55 (96.5)	1 (1.8)	0 (0.0)	0 (0.0)	8 (14.0)
Antipyretic analgesics	8.0 (4.9, 12.3)	5.0 (4.0, 5.5)	2.4 (0.5, 5.0)	2 (4.3)	44 (95.7)	0 (0.0)	0 (0.0)	0 (0.0)	2 (4.3)
Neurological agents	14.1 (7.1, 25.6)	5.0 (4.0, 7.0)	3.7 (1.4, 5.5)	3 (10.0)	27 (90.0)	0 (0.0)	0 (0.0)	0 (0.0)	3 (10.0)
Cardiovascular agents	11.5 (7.1, 18.6)	6.0 (4.0, 9.0)	1.0 (0.4, 5.2)	0 (0.0)	29 (100.0)	0 (0.0)	0 (0.0)	0 (0.0)	8 (27.6)
Pesticides	9.9 (5.4, 14.2)	9.0 (5.0, 13.3)	7.7 (3.1, 12.4)*	2 (1.8)	102 (89.5)	7 (6.1)	2 (1.8)	1 (0.9)*	2 (1.8)*
Herbicides	9.9 (5.8, 14.2)	10.0 (6.8, 15.0)	10.3 (5.6, 14.5)	1 (1.3)	67 (87.0)	6 (7.8)	2 (2.6)	1 (1.3)	1 (1.3)
Insecticides	7.4 (3.4, 11.1)	5.0 (5.0, 8.3)	5.0 (0.8, 7.5)	1 (4.3)	21 (91.3)	1 (4.3)	0 (0.0)	0 (0.0)	0 (0.0)
Rodenticide	10.7 (5.3, 16.3)	4.5 (3.8, 6.3)	3.0 (0.4, 5.5)	0 (0.0)	13 (100.0)	0 (0.0)	0 (0.0)	0 (0.0)	1 (7.7)
Multi-category mixed	10.9 (6.8,14.7)	6.0 (4.0, 6.0)	0.6 (0.4, 3.5)	1 (3.0)	32 (97.0)	0 (0.0)	0 (0.0)	0 (0.0)*	4 (12.1)
Industrial chemicals	11.4 (4.9, 14.2)	6.0 (3.0, 8.0)	4.5 (1.5, 7.6)	1 (5.9)	15 (88.2)	0 (0.0)	1 (5.9)	0 (0.0)	3 (17.6)
Household chemicals	6.9 (4.8, 11.8)	11.0 (7.5, 20.0)	0.4 (0.2, 3.7)	0 (0.0)	10 (100.0)	0 (0.0)	0 (0.0)	0 (0.0)	0 (0.0)
Ethanol	5.0 (2.9, 6.7)	2.5	0.2 (0.2, 0.3)*	1 (6.2)	15 (93.8)	0 (0.0)	0 (0.0)	0 (0.0)	0 (0.0)
Other toxic agents	6.3 (0.3, 9.5)	2.0 (2.0, 4.0)	1.2 (0.4, 2.3)	1 (10.0)	9 (90.0)	0 (0.0)	0 (0.0)	0 (0.0)	1 (10.0)

*p < 0.05 for the major category of poisoning compared to pharmaceuticals.

Clinical outcomes also varied significantly by categories (*p* < 0.001). While 98.1% of cases had favorable outcomes, pesticide exposures showed higher rates of unfavorable outcomes than pharmaceuticals (*p* < 0.001). Pesticides accounted for 81.8% (9/11) of all unfavorable outcomes. Herbicide poisonings caused 8 cases of deterioration or mortality (10.4%), with 1 case (4.3%) from insecticides. Fatalities resulted from paraquat (*n* = 2) and nitrite (*n* = 1; Table 4).

Among the patients, 110 (12.0%) had recurrent poisoning. Recurrent poisoning was significantly less frequent in the pesticide group than in the pharmaceutical group (*p* < 0.05; Table 4).

### Factors associated with hospitalization requirement

Among 915 adolescent acute poisoning cases, 474 (51.8%) met the composite criteria for hospitalization. In the fully adjusted binary logistic regression model, pesticide exposure (reference: pharmaceuticals) was the strongest predictor of hospitalization (aOR 4.55, 95% CI 2.63–8.22; *p* < 0.001), followed by industrial chemical poisoning (aOR 3.98, 95% CI 1.33–14.85; *p* = 0.021). Multi-category exposures were associated with reduced hospitalization risk (aOR 0.41, 95% CI 0.18–0.89; *p* = 0.031). Intentional self-harm (reference: substance abuse) showed 3.7-fold higher odds of hospitalization (aOR 3.69, 95% CI 1.40–11.33; *p* = 0.013), and preexisting mental illness increased odds by 51% (aOR 1.51, 95% CI 1.09–2.10; *p* = 0.015). Prehospital interventions were associated with higher hospitalization odds (aOR 1.75, 95% CI 1.29–2.38; *p* < 0.001), while each year increase in age showed 20% decreased odds (aOR 0.80, 95% CI 0.73–0.87; *p* < 0.001) (Table 5).

**Table 5:** Independent predictors of hospitalization for adolescent acute poisoning identified by multivariable logistic regression.

Factors	ED observation only (*n*.)	Hospitalization required (*n*.)	Coefficient	*p-*value	aOR (95%CI)
Sex (male)	117	135	0.14	0.421	1.15 (0.82, 1.61)
Age (y.)	477	474	−0.22	<0.001	0.80 (0.73, 0.87) ^*^
Time from poisoning to hospital arrival (h.)	477	474	0.01	0.255	1.01 (1.00, 1.02)
Prehospital interventions	240	344	0.56	<0.001	1.75 (1.29, 2.38) ^*^
Preexisting comorbidities					
Mental illness	267	283	0.41	0.015	1.51 (1.09, 2.10) ^*^
Chronic disease	10	13	0.45	0.364	1.56 (0.60, 4.16)
Categorization of toxic substances					
Pesticides	19	95	1.52	<0.001	4.55 (2.63, 8.22) ^*^
Multi-category mixed	24	9	−0.88	0.031	0.41 (0.18, 0.89) ^*^
Industrial chemicals	4	13	1.38	0.021	3.98 (1.33, 14.85) ^*^
Household chemicals	7	3	−0.76	0.308	0.47 (0.09, 1.89)
Ethanol	14	2	−0.21	0.809	0.81 (0.11, 3.89)
Other toxic agents poisoning	5	5	−26.20	0.974	0.00, (NA, 5.70E+22)
Intent of poisoning					
Suicidal	408	457	1.31	0.013	3.69 (1.40, 11.33) ^*^
Misuse	0	9	42.93	0.967	4.39E+18 (0.00, NA)
Environment	2	1	13.31	0.982	6.05E+05 (0.00, NA)
Other	3	1	2.84	0.620	17.19 (0.02, 9731.22)
Gastrointestinal decontamination	224	186	0.17	0.264	1.19 (0.88, 1.61)
Recurrent poisoning episodes	57	53	−0.02	0.941	0.98 (0.64, 1.52)

*p < 0.05, ED: Emergency department; aOR: adjusted odds ratio; CI: confidence interval; Reference groups: Pharmaceuticals (toxic substances), Substance abuse (poisoning intent), No intervention (prehospital care), No comorbidities (chronic/mental disease); Extreme OR values reflect quasi-complete separation in logistic regression.

## Discussion

This retrospective cohort study analyzed epidemiological patterns of acute poisoning among adolescents from a major tertiary poisoning referral center in Beijing, China. To our knowledge, it is the first large-scale study to provide a comprehensive characterization of acute adolescent poisoning in this metropolitan area.

The findings reveal age-related vulnerabilities. An Italian study reported a mean age of 15.8 ± 2.0 years in their adolescent acute poisoning cohort (6), while French data indicated a peak incidence in pediatric poisoning at age 13 for males and 14 for females (7). Our cohort exhibited similar peaks at age 15 for males and 14 for females, suggesting common psychosocial stressors during adolescence across cultures. In China, the 14–16-year age group coincides with secondary school entrance examinations. Academic pressure combined with ongoing neurocognitive development may increase the risk of intentional self-poisoning among adolescents with poor coping mechanisms. Females constituted 72.5% of cases, aligning with reports from both developed and developing countries over the past decade (1, 8–18). This confirms the association between female sex and heightened vulnerability to deliberate self-harm among adolescents (19, 20), highlighting the need for sex-specific mental health interventions targeting adolescent females. While the deep-seated cultural and psychosocial drivers (e.g., gendered social expectations, family dynamics) warrant dedicated investigation, such an analysis extends beyond the descriptive scope of this study and points to a crucial direction for future research.

Oral ingestion was the primary exposure route, with intentional self-harm accounting for the overwhelming majority (94.5%) of cases, which emerged as the predominant pattern of poisoning.in our cohort. Neuropsychiatric disorders were documented in 60.1% of patients, consistent with studies linking self-poisoning to psychiatric conditions (6, 13, 15, 17). Immaturity in prefrontal-limbic circuitry and adolescent psychosocial challenges may increase suicide risk (21–23). Neuropsychiatric disorders represent a key risk factor, with female sex, adolescence, and prior psychiatric care as predictors of self-harm (24). These findings, driven by this predominance of self-harm, underscore the need for mental health promotion and early intervention for at-risk adolescents.

Urban residents accounted for a higher proportion of cases (58.9%) compared to rural residents (22.6%), consistent with patterns in Iran and Zambia (17, 18). This disparity may reflect that in urban areas, while toxic agents (particularly pharmaceuticals) are more readily available, there is also faster access to medical care after exposure. Urban cases also showed greater female predominance than rural cases, possibly influenced by academic and familial pressures. These findings support implementing geographically tailored, gender-sensitive prevention strategies. For instance, urban-focused efforts should prioritize secure medication storage, mental health literacy in schools, and accessible adolescent counseling services. In rural areas, prevention must address the high lethality of pesticide exposures through safer storage practices, community education on early recognition of poisoning, and strategies to reduce pre-hospital delays in seeking care.

Seasonal patterns showed distinct variations: incidence peaked in autumn and was lowest in winter. The September–October surge, particularly among females, aligns with academic transitions (e.g., selective high school entrance), where scholastic pressure may elevate poisoning risk, consistent with U.S. data (12). This timing underscores the critical need for proactive mental health screening and support within schools at the start of the academic year. Exposures peaked at 18:00–24:00, followed by 12:00–18:00, matching global reports (11, 25). Circadian-mediated emotional dysregulation during these hours may increase deliberate self-poisoning risk. Enhanced supervision during high-risk periods could help mitigate this vulnerability. Practical measures may include structured evening activities, increased family engagement during these hours, and ensuring real-time accessibility of crisis support hotlines.

Pharmaceuticals were the predominant toxic agents, with antipsychotics and antidepressants as main subclasses. Pesticides ranked second, mainly herbicides such as paraquat and diquat. Pharmaceutical exposures were more frequent in females, whereas pesticides, industrial/household chemicals, ethanol, and other toxins were more common in males. Urban areas showed higher pharmaceutical prevalence (63.6%), while rural areas had more pesticide poisonings (53.8%). These patterns align with Chinese and global reports (5, 11, 13, 14, 16–18), necessitating context-specific interventions like mental health access, medication regulation, and pesticide safety education. The low incidence of acute ethanol intoxication in this clinical cohort contrasts with higher prevalence reported in community-based surveys or emergency settings in regions with different drinking cultures (1, 26), likely reflecting our study’s focus on severe, referral-based poisonings within a specific socio-regulatory context. This pattern is consistent with reports from other regions in China (5, 27). Narcotics-related poisonings were rare (0.2%), contrasting with Iran (30.8%) (17), Egypt (6%) (14), and U.S. fentanyl overdoses (8), likely due to China’s stringent narcotics control policies.

Regional disparities existed in adolescent poisoning management. The ICU admission rate (10.3%) exceeded Rome’s 7.9% (6), suggesting greater poisoning severity in our cohort. Despite guidelines discouraging routine gastric lavage or induced emesis (28), gastrointestinal decontamination occurred in over 50% of cases, consistent with other regions where 45.8% received guideline-concordant decontamination (29). The variation in extracorporeal elimination rates— 22.3% in our cohort versus 0.7% reported from Rome (6) — is likely attributable to the higher proportion of severe pesticide and industrial chemical poisoning in our region, as well as differences in institutional protocols (30), highlighting the need for multicenter trials to formulate standardized detoxification strategies.

Compared with pharmaceutical poisonings, pesticide exposure was associated with higher general ward and ICU admission rates. These cases also required more frequent gastrointestinal decontamination, antidote administration, extracorporeal elimination, and mechanical ventilation, along with longer treatment durations. A similar pattern was observed for industrial chemical exposures, indicating both most pesticides and industrial chemicals cause greater toxicity. In contrast, ethanol intoxication showed lower hospitalization and ICU admission rates and shorter treatment duration, consistent with its low acute toxicity. These characteristics, supported by subclass analyses, align with the inherent toxicological and toxicokinetic properties of the substances. Among pesticide subclasses, herbicide exposures (e.g., paraquat and diquat) were associated with higher hospitalization and ICU admission rates compared to insecticides and rodenticides, likely due to their higher intrinsic toxicity (31).

Overall outcomes were largely favorable, although 1.2% of patients experienced unfavorable outcomes. The mortality rate observed in this study exceeded reported global adolescent poisoning mortality rates (0–0.7%) (8, 9, 12, 14, 15, 17, 18). U.S. 2023 data indicated that analgesics, chemicals, antidepressants, and cardiovascular agents were the primary causes of poisoning-related mortality in adolescents, with no documented pesticide-related fatalities in this population (8). In contrast, diquat and paraquat poisonings were associated with high mortality in this region. The accessibility of these toxic agents has increased through online e-commerce platforms, elevating the risk of intentional and accidental exposure. These findings highlight the need for stricter regulatory measures — including controlled distribution and sales restrictions — as well as coordinated public health campaigns to reduce adolescent poisoning mortality.

Multivariable analysis identified independent predictors of hospitalization requirement: younger age, prehospital interventions, preexisting neuropsychiatric disorders, pesticide exposure, multi-category exposures, industrial chemical poisoning, and intentional self-harm. These align with Brazilian pediatric poisoning data linking hospitalization to high-lethality agents and intentional self-harm (32). Two universal determinants of severity were identified: exposure to highly toxic agents (such as herbicides) and deliberate self-poisoning, both predicting the need for intensive clinical interventions. Notably, prehospital interventions were associated with 1.75-fold higher hospitalization odds, likely because severe cases more frequently received prehospital care.

Several limitations of this study should be acknowledged. First, the retrospective design is susceptible to selection bias, and some patients were excluded owing to incomplete medical records. This inconsistency also prevented the systematic use of a standardized poisoning severity score (e.g., PSS). Second, the data were derived from a single tertiary referral center. This setting may inherently select for more severe cases, which should be considered when extrapolating findings to all community-level adolescent poisoning incidents. Nevertheless, as the largest poisoning treatment center in Beijing — receiving referrals from the city and surrounding regions — our cohort provides a valid epidemiological profile of poisoning cases severe enough to reach higher-level care in this area. Third, the investigation period (2021–2023) coincided with the COVID-19 pandemic, during which public health control measures may have influenced healthcare-seeking behavior.

## Conclusion

Acute poisoning among adolescents in Beijing and surrounding regions demonstrates a significant female predominance, with a peak incidence observed between ages 14 and 15. Most cases were associated with preexisting neuropsychiatric disorders, and psychotropic medications represented the most common toxic agents. Pesticide exposures exhibited the highest case-fatality rate. Key prevention strategies should include strengthening psychological support, enforcing stricter regulation of psychotropic medications, and restricting access to highly toxic pesticides to reduce incidence. Additionally, integrated toxicological and clinical research is essential to develop effective antidotes and treatment protocols for lowering mortality.

## Data Availability

The original contributions presented in the study are included in the article/supplementary material, further inquiries can be directed to the corresponding authors.
